# Supplementary material for: Tungsten-Doped VO2/Starch Derivative Hybrid Nanothermochromic Hydrogel for Smart Window
Source: Nanomaterials (Basel). 2019 Jul 2;9(7):970. doi: 10.3390/nano9070970 (PMC6669878; doi:10.3390/nano9070970)
Supplement: Supplementary file 1 [file nanomaterials-09-00970-s001.pdf]

Supplementary Information

Tungsten-Doped VO_2/Starch Derivative Hybrid Nanothermochromic Hydrogel for Smart Window

Yu Wang [1], Fang Zhao [1,*], Jie Wang [1,*], Li Li [1], Kaiqiang Zhang [2], Yulin Shi [2], Yanfeng Gao [3,4,*] and Xuhong Guo [1,2,*]

[1] State Key Laboratory of Chemical Engineering, East China University of Science and Technology, Shanghai 200237, China

[2] Engineering Research Center of Materials Chemical Engineering of Xinjiang Bingtuan, Key Laboratory of Materials Chemical Engineering of Xinjiang Uygur Autonomous Region, Shihezi University, Shihezi 832000, China

[3] School of Materials Science and Engineering, Shanghai University, Shanghai 200444, China

[4] School of Materials Science and Energy Engineering, Foshan University, Foshan 528000, China

* Correspondence: Fzhao1@ecust.edu.cn (F.Z.); jiewang2010@ecust.edu.cn (J.W.); yfgao@shu.edu.cn (Y.G.); guoxuhong@ecust.edu.cn (X.G.); Tel.: +86-21-64253488 (F.Z.); +86-21-64253491 (J.W. & X.G.); +86-21-66138005 (Y.G.)

1. X-ray diffraction pattern and phase transition behavior of VO_2(M)

The XRD spectra of monoclinic VO_2(M) (JCPDS card No. 43-1051) and W-VO_2(M) were given in Fig. S1. According to our previous work [1], even when W content was increased to 1.0%, only a slight shift of the M (011) peak was observed for W-doped VO_2(M) samples, which was also confirmed in our experiments. Both XRD results exhibit monoclinic crystal phase without any shift observed. The DSC result clearly shows that the phase transition temperature of W-doped VO_2(M) was reduced from 68 to 39 °C which is close to room temperature and LCST (lower critical solution temperature) of HBPS.

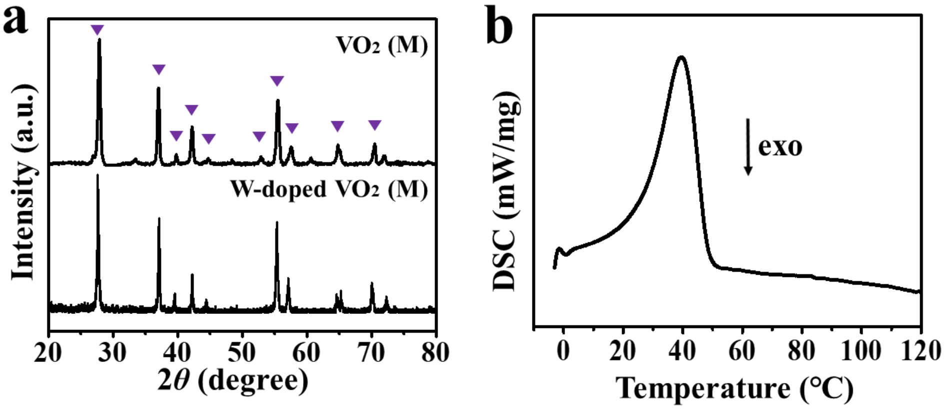

Figure S1. (a) XRD results of the VO_2(M) NPs and the W–VO_2(M) NPs. (b) DSC curve as function of temperature upon heating of W-doped VO_2(M) NPs.

2. Description of the different organic-inorganic hybrid samples

The actual contents of V and W-doped VO_2(M) in the composite film are shown in Table S1.

Table S1. Description of the two different organic-inorganic hybrid thermochromic films investigated in this work.

Sample	C_{HBPS-1} [a]	C_V [b]	$C_{W-doped\ VO2(M)}$ [c]
Composite-1	5 g/L	14.85 mg/L	24.18 mg/L
Composite-2	5 g/L	47.05 mg/L	76.62 mg/L
Composite-3	5 g/L	77.67 mg/L	126.48 mg/L

[a] The actual HBPS-1 NP content in the hybrids;.[b] The actual V content in the hybrids determined by ICP;.[c] The actual content of W-doped VO_2(M) calculated from C_V.

3. Morphology of W-doped VO_2 in Composite-2

To obtain a uniform hybrid of W-doped VO_2/starch derivative, zirconia pellet with 0.5 mm diameter was used for grinding to achieve the deaggregation of W-doped VO_2. As shown in the insert picture of Fig. S2a. the zirconia pellet was added into W-doped VO_2 suspension, and the mixture was stirred for 24 hours. Fig. S2a shows that the hydraulic radius of W-doped VO_2 before and after stirring was reduced from 278.3 nm to 98.7 nm. Then, the zirconia pellet was filtered out and the resulted W-doped VO_2 nanosuspension was directly dispersed into the starch derivative matrix. As shown in Fig. S2b, the W-doped VO_2 was well dispersed in the starch derivatives and the diameter of the W-doped VO_2 was 20-60 nm.

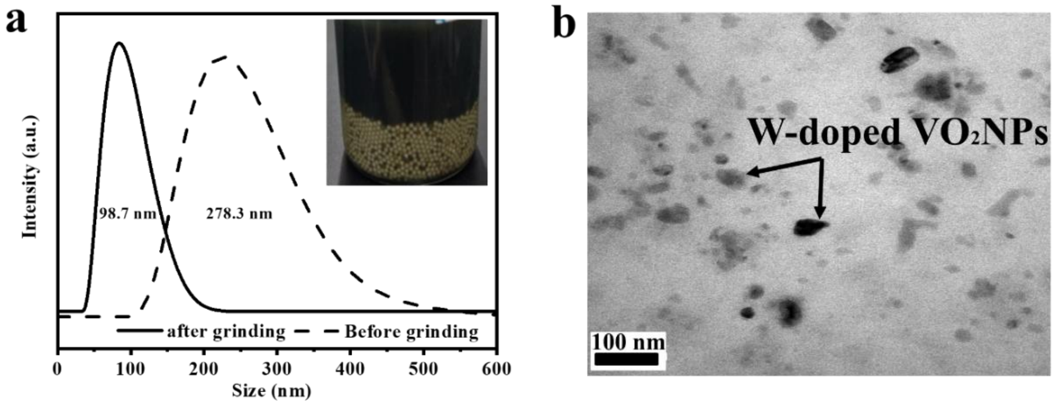

Figure S2. (a) Diameter of W-doped VO_2 NPs before (dash line) and after (solid line) grinding, and the insert picture shows the W-doped VO_2 suspension with white zirconia pellets. (b) TEM image of Composite-2.

4. Hysteresis loop of Composite-2

The transmittance at a fixed wavelength of 550 nm in the temperature range 20 to 45 °C for Composite-2 was recorded in a heating and cooling cycle and plotted versus temperature in Fig. S3. It can be seen that the hysteresis loop for Composite-2 had a relatively narrow loop gap. And the cloud point temperature of composite-2 was determined to be about 33 °C for the cooling and heating cycle with a T_{lum} decrease from 96% to 17%.

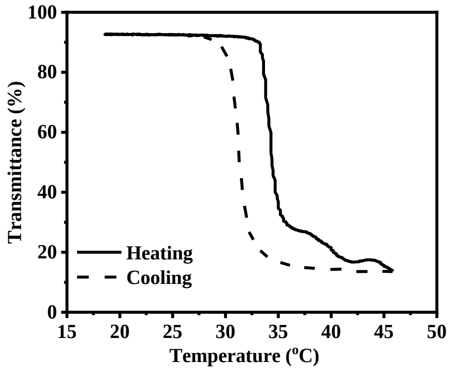

Figure S3. Hysteresis loop for the temperature-dependent transmittance of the Composite-2 at a wavelength of 550 nm.

5. Comparison of optical performance between previously reported smart window system and our system

As shown in Table S1, comparing with the result of the literature listed, our composite film shows excellent solar modulation properties (ΔT_{sol} = 34.3%) with high luminous transmittance ($T_{lum, average}$ = 78.7%). Also, the high $T_{lum, average}$ value of our composite indicates that we do not need extra energy to maintain good visibility indoors.

Table S2. Comparison of my hybrid sample with other thermochromic materials (note: "–" means data not available).

Category			Modulated solar spectrum (nm)	T_{lum} (%)	ΔT_{sol} (%)	Ref.
Thermal-responsive materials	VO_2	VO_2	700-2500	45.6	22.3	2
		W + Zr-doped VO_2	700-2500	56.4	12.3	3
	Hydrogels	PNIPAm	250-2200	70.7	25.5	4
		Modified cellulose	250-2200	67.4	25.7	5
		Modified starch				
			250-2200	87.7	40.1	
	Metamaterials	Kiri-Kirigami				6
			200-2200	-	-	
		metamaterials				7
	Perovskites	Halide perovskite				
			400-700	-	-	
	Liquid crystals					8
			400-2200	-	-	
						9
	Non-	VO_2/PU				
VO_2 based hybrid			700-2500	45.6	22.3	
	responsive	VO_2/Si-Al gel				2
thermochromic			700-2500	59.1	12.0	
		VO_2/PDMS				10
material			700-2500	85.0	-	
		VO_2/TiO_2				11
			700-2500	61.2	14.6	
		VO_2/PNIPAm				12
			250-2500	62.6	34.7	
	responsive	VO_2/HPC				13
			250-2500	56.0	36.0	
		VO_2/NLETS				14
			500-2500	71.0	18.2	
		$VO_2@SiO_2$/PNIPAm				15
			250-2500	75.6	62.7	
		Our composite				16
			250-2500	78.7	34.3	
		VO_2/Statch				

6. References

1. Shen, N.; Dong, B.; Cao, C.; Chen, Z.; Liu, J.; Luo, H.; Gao,Y.; Lowered phase transition temperature and excellent solar heat shielding properties of well-crystallized VO_2 by W doping, Phys. Chem. Chem. Phys. 2016, 18, 28010-28017.
2. Chen, Z.; Gao, Y.; Kang, L.; Cao, C.; Chen, S.; Luo, H.; Fine crystalline VO_2 nanoparticles: synthesis, abnormal phase transition temperatures and excellent optical properties of a derived VO_2 nanocomposite foil, J. Mater. Chem. A. 2014, 2, 2718-2727.
3. Shen, N.; Chen, S.; Chen, Z.; Liu, X.; Cao, C.; Dong, B.; Luo, H.; Liu, J.; Gao, Y.; The synthesis and performance of Zr-doped and W–Zr-codoped VO_2 nanoparticles and derived flexible foils, J. Mater. Chem. A. 2014, 2, 15087-15093.
4. Zhou, Y.; Cai, Y.; Hu, X.; Long, Y.; Temperature-responsive hydrogel with ultra-large solar modulation and high luminous transmission for "smart window" applications, J. Mater. Chem. A. 2014, 2, 13550-13555.
5. Yang, Y.; Zhou, Y.; Chiang, F.; Long, Y.; Tungsten doped VO_2/microgels hybrid thermochromic material and its smart window application, RSC Adv. 2017, 7, 7758-7762.
6. Zhang, K.; Shi, Y.; Wu, L.; Chen, L.; Wei, T.; Jia, X.; Chen, Z.; Li, M.; Xu, Y.; Wang, Y.; Gao, Y.; Guo, X.; Thermo- and pH-responsive starch derivatives for smart window, Carbohydr. Polym. 2018, 198, 209-216.
7. Tang, Y.; Lin, G.; Yang, S.; Yi, Y.; Kamien, R.; Yin, J.; Programmable Kiri- Kirigami Metamaterials, Adv. Mater. 2017, 29, 604262.
8. lin, J.; Lai, M.; Dou, L.; Kley, C.; Chen, H.; Peng, F.; Sun, J.; Lu, D.; Hawks, S.; Xie, C.; Cui, F.; Alivisatos, A.; Limmer, D.; Yang, P.; Thermochromic halide perovskite solar cells, Nat. Mater. 2018, 17, 261-267.
9. Liang, X.; Guo, S.; Chen, M.; Li, C.; Wang, Q.; Zou, C.; Zhang, C.; Zhang, L.; Guo, S.; Yang, H.; A temperature and electric field-responsive flexible smart film with full broadband optical modulation, Mater. Horiz. 2017, 4, 878-884.
10. Liu, C.; Cao, X.; Kamyshny, A.; Law, J.; Magdassi, S.; Long, L.; VO_2/Si-Al gel nanocomposite thermochromic smart foils: largely enhanced luminous transmittance and solar modulation, J. Colloid Interface Sci, 2014, 427, 49-53.
11. Moot, T.; Palin, C.; Mitran, S.; Cahoon, J.; Lopez, R.; Designing plasmon-enhanced thermochromic films using a vanadium dioxide nanoparticle elastomeric composite, Adv. Opt. Mater. 2016, 4, 578-583.
12. Chen, Z.; Cao, C.; Chen, S.; Luo, H.; Gao, Y. Crystallised mesoporous TiO_2(A)–VO_2(M/R) nanocomposite films with self-cleaning and excellent thermochromic properties, J. Mater. Chem. A. 2014, 2, 11874-11884.
13. Zhou, Y.; Cai, Y.; Hu, X.; Long, Y.; VO_2/hydrogel hybrid nanothermochromic material with ultra-high solar modulation and luminous transmission, J. Mater. Chem. A. 2015, 3, 1121-1126.
14. Yang, Y.; Zhou, Y.; Chiang, F.; Long, Y.; Tungsten doped VO_2/microgels hybrid thermochromic material and its smart window application, RSC Adv. 2017, 7, 7758-7762.
15. Zhu, J.; Huang, A.; Ma, H.; Chen, Y.; Zhang, S.; Ji, S.; Bao, S.; Jin, P.; Hybrid films of VO_2 nanoparticles and a nickel(ii)-based ligand exchange thermochromic system: excellent optical performance with a temperature responsive colour change, New J. Chem. 2017, 41, 830-835.
16. Wang, Y.; Zhao, F.; Wang, J.; Khan, A.; Shi, Y.; Chen, Z.; Zhang, K.; Gao, Y.; Guo, X.; VO_2@SiO_2/Poly(N-isopropylacrylamide) Hybrid Nanothermochromic Microgels for Smart Window, Ind. Eng. Chem. Res. 2018, 57, 12801-12808.
